# Revisiting Mt Fuji’s groundwater origins with helium, vanadium and environmental DNA tracers

**DOI:** 10.1038/s44221-022-00001-4

**Published:** 2023-01-19

**Authors:** O. S. Schilling, K. Nagaosa, T. U. Schilling, M. S. Brennwald, R. Sohrin, Y. Tomonaga, P. Brunner, R. Kipfer, K. Kato

**Affiliations:** 1 Hydrogeology, Department of Environmental Sciences, University of Basel, Basel, Switzerland; 2 Department Water Resources and Drinking Water, Eawag–Swiss Federal Institute of Aquatic Science and Technology, Dübendorf, Switzerland; 3 Centre for Hydrogeology and Geothermics, Université de Neuchâtel, Neuchâtel, Switzerland; 4 Department of Geosciences, Shizuoka University, Shizuoka, Japan; 5 Department of Geology and Geological Engineering, Université Laval, Quebec, Quebec Canada; 6 Entracers GmbH, Dübendorf, Switzerland; 7 Institute of Biogeochemistry and Pollutant Dynamics and Institute of Geochemistry and Petrology, Swiss Federal Institute of Technology Zurich (ETHZ), Zurich, Switzerland

**Keywords:** Hydrology, Geodynamics, Geochemistry, Microbial ecology, Volcanology

## Abstract

Known locally as the water mountain, for millennia Japan’s iconic Mt Fuji has provided safe drinking water to millions of people via a vast network of groundwater and freshwater springs. Groundwater, which is recharged at high elevations, flows down Fuji’s flanks within three basaltic aquifers, ultimately forming countless pristine freshwater springs among Fuji’s foothills. Here we challenge the current conceptual model of Fuji being a simple system of laminar groundwater flow with little to no vertical exchange between its three aquifers. This model contrasts strongly with Fuji’s extreme tectonic instability due to its unique location on top of the only known continental trench–trench–trench triple junction, its complex geology and its unusual microbial spring water communities. On the basis of a unique combination of microbial environmental DNA, vanadium and helium tracers, we provide evidence for prevailing deep circulation and a previously unknown deep groundwater contribution to Fuji’s freshwater springs. The most substantial deep groundwater upwelling has been found along Japan’s most tectonically active region, the Fujikawa-kako Fault Zone. Our findings broaden the hydrogeological understanding of Fuji and demonstrate the vast potential of combining environmental DNA, on-site noble gas and trace element analyses for groundwater science.

## Abstract (web summary)

Mt Fuji’s freshwater springs were believed to be fed exclusively by shallow groundwater aquifers. Using a newly developed combination of tracer techniques, this study finds widespread vertical exchange between shallow and deeper aquifers and shows evidence of a substantial deep groundwater contribution to the springs.

## Main

**Fig. 1:**
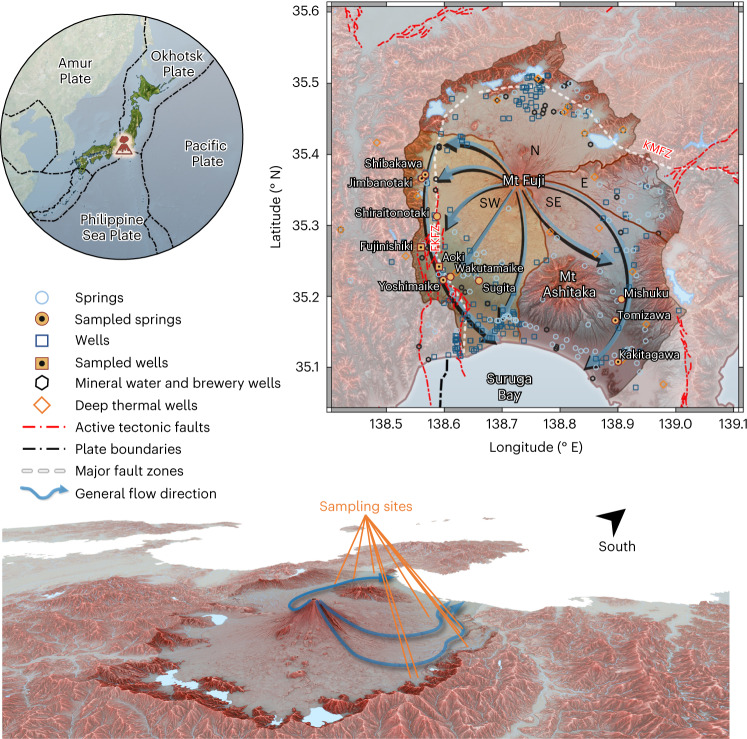
Fuji catchment. Top left: Fuji’s location on the trench–trench–trench triple junction between the Amur, Okhotsk, and Philippine Sea plates in central Japan. Top right: map of Fuji catchment, its four sub-basins (with the southwestern sub-basin highlighted in yellow), the general groundwater flow directions of the southwestern and southeastern sub-basins, the major fault zones, the currently active tectonic faults, the sampled sites and all data points obtained in this study or gathered from literature and the national groundwater database of Japan. Black dots in the symbols for sampled sites indicate the locations of eDNA analyses. Bottom: 3D map of Mt Fuji oriented towards the southeast. Fuji catchment is highlighted, and the sampling sites and general flow directions of the southwestern and southeastern sub-basins are indicated. KMFZ = Kotsu-Matsuda Fault Zone. Coordinate reference system: WGS 84/Pseudo-Mercator. Composite map sources: satellite imagery^161^; digital elevation model^162^; red 3D hillshade map^163,164^; active tectonic fault locations^165^; plate boundaries and major tectonic faults^43,166,167^.

With its near-perfect conical shape, Japan’s volcanic Mt Fuji (3,776 m above sea level (ASL)) may arguably be the world’s best-known mountain^1^. Known locally as the water mountain, for millennia Fuji has provided safe drinking water to millions of people via its abundant groundwater and groundwater-fed springs. The abundance of freshwater resources arises from large amounts of precipitation that occur due to Fuji’s proximity to the Pacific Ocean and Sea of Japan, and its unique location on top of Fuji triple junction, the only known continental trench–trench–trench triple junction on Earth^2–4^ (Fig. 1). Due to this unique geologic setting, Fuji consists primarily of basalt and is much more permeable than other arc stratovolcanoes, which are mostly composed of poorly permeable andesitic magmas^5–13^. Owing to its long passage through basalt^14^, Fuji’s groundwater is very soft and strongly enriched in vanadium, making Fuji’s rivers the most vanadium enriched on Earth^15–17^. Fuji is so important that it has UNESCO World Heritage Site status^18^, with multiple springs designated as national Natural Monuments^19–21^.

On top of the ever-increasing demand for water by residents, tourists, industry and agriculture, a microcosm of premium food industries has developed, producing goods that depend heavily on Fuji’s clean water. Japan’s largest green tea plantation area on the southern slopes and large whisky distilleries on the eastern slope can only operate because of the consistently large supply of soft, high-quality groundwater. With increasing success, numerous water bottling companies now sell vanadium-rich groundwater pumped from deep below Fuji as healthy mineral water^22–24^. Moreover, it was found that if vanadium-rich water is used in sake (*nihonshu*) brewing, undesired stale aroma compounds are suppressed while the desired sweet taste is fostered^25,26^, potentially explaining the award-winning international success of Fuji’s sake breweries^27,28^.

Although Fuji has been studied extensively and its complex geology is well documented, water quality and quantity are declining and many hydrogeological questions remain unsolved^29–35^. According to current understanding, of the 2.2 km^3^ (or 2,500 mm) of precipitation per year, 90% form groundwater recharge^7,36^. After 15–40 yr (ref. 14), 1.7 km^3^ emerge each year at the foothills as springs, rivers and lakes, while the rest leaves the catchment as groundwater^7^. Although groundwater also flows through the deep Ko (‘old’)-Fuji aquifer, formed during the older Hoshiyama volcanic stage (100–17 kyr ago (ka)), springs are believed to be fed exclusively from the shallower Shin (‘new’)-Fuji and Surficial aquifers, originating from the younger Fujinomiya and Subashiri stages (<17 ka)^7,13,17,37,38^. Except for some seepage^31^, vertical exchange between the deep and shallow aquifers is currently considered to be negligible^7,17,36,39^. This simple model, however, is at odds with Fuji’s complex geology and fails to explain the decline in water quality^29,30,40–42^.

Here we present evidence for a substantial contribution of Ko-Fuji deep groundwater to the springs along Fuji’s Fujikawa-kako Fault Zone (FKFZ), Japan’s most tectonically active region (Fig. 1)^10,43–45^. Our environmental DNA (eDNA), helium (He) and vanadium (V) measurements, together with a compilation of hydrochemical data from many earlier studies, broaden our hydrogeological understanding of Fuji and demonstrate the vast potential of combining eDNA, on-site noble gas and trace element analyses for groundwater science.

## Shared meteoric origin and the limits of classic tracers

Despite its critical inability to fully explain the recent water quality decline, the simplistic conceptual model of laminar groundwater flow within Fuji is still accepted. The culprits are the classic methods that have been applied to understand Fuji’s hydrogeology so far, namely shallow groundwater levels, major ions and stable water isotopes. These classic hydrogeological methods are the most widely employed and are typically used to identify groundwater flow directions, recharge zones and seasonality, and flow through distinct hydrochemical zones. However, in mountainous systems where shallow groundwater levels follow the topography, where physical mixing between different water types is prevalent and the major ion compositions of different water types are very similar, these classic methods often do not allow an unambiguous assessment of groundwater recharge and flow paths^36,46,47^.

**Fig. 2:**
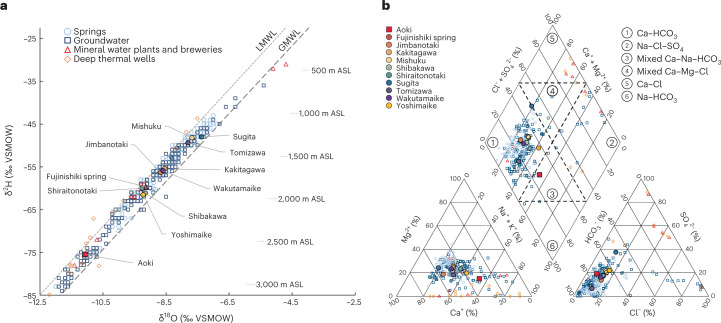
Overview of the stable water isotope and major ion compositions of springs and groundwater in Fuji catchment. **a**, Stable water isotope composition of investigated sites alongside all available measurements of springs, groundwater wells, mineral water and brewery wells and thermal deep wells; stable water isotope-derived recharge elevations after Yasuhara et al.^36^ are indicated. Local meteoric water line (LMWL)^36^: δ^2^H = 8 × δ^18^O + 15.1‰. Global meteoric water line (GMWL)^51^: δ^2^H = 7.93 × δ^18^O + 8.99‰. VSMOW, Vienna standard mean ocean water. **b**, Piper plot of the major ion composition of all available data on springs, groundwater wells, mineral water and brewery wells, and thermal deep wells. The fields of hydrochemical water types are separated by dashed lines and marked by numbered circles (see legend). Data points in **a** and **b** represent site-averaged values. All individual data points and corresponding references are provided in tabular form in Supplementary Data 1.

As the hydrogeology of Fuji is dominated by elevational gradients, shallow groundwater levels follow the general topography and provide no indication of vertical interactions between different aquifers^17,39,48,49^. The Ko-Fuji deep aquifer is known to be confined and artesian, but the pressure distribution is unknown^17,29–31,39^. Stable water isotope signatures (i.e., δ^2^H & δ^18^O) of streams, springs and groundwater fall between the local and the global meteoric water lines (Fig. 2a and Supplementary Section 1), revealing a common meteoric origin and uniform evaporation effect^36,50^, but recharge elevation, seasonality and physical mixing cannot be separated on the basis of those signals^51^.

Springs and groundwaters around Fuji are all cold (~14.5 °C), fresh (~400 μS cm^−1^), mildly alkaline (~7.75 pH) and classified as Ca–HCO_3_ type. Along Suruga Bay, the Na–Cl–SO_4_ type prevails due to local seawater intrusion^17,32,41,42^ (Fig. 2b). Only deep thermal groundwaters, which are pumped from Fuji’s basement from a depth of 1,500 m and used in local spas (*onsen*), are mildly warm (~40 °C), slightly alkaline (~8.75 pH) and mineralized (~1 g l^−1^). Due to the great depth and admixture of seawater inclusions from the green tuff of Fuji’s basement, this thermal water is classified as Ca–Cl and Na–Cl–SO_4_^52,53^. However, besides the seawater inclusions in Fuji’s basement and seawater intrusion along Suruga Bay, all natural waters in Fuji catchment are of meteoric origin^52,53^. The shared meteoric origin, large elevational range and hydrochemical similarity of the natural waters mean that the classic tracer methods employed are of limited applicability for differentiating water bodies/components. Rather than provoking further research, these observations were widely accepted and interpreted in a straightforward manner as being the result of a laminar flow system. However, this interpretation obscures potentially existing vertical interactions between the different aquifers, and instead fosters the idea of Fuji being a relatively simple groundwater system.

## Deep groundwater upwelling revealed by He, V and eDNA

To overcome the limitations of the classic methods applied in Fuji catchment so far, and to critically assess the governing conceptual hydrogeological model of Fuji, we carried out a multi-tracer investigation combining three unconventional and new tracer methods: on-site and laboratory-based He, V and eDNA analyses. Below we report the major findings for each tracer, focusing on the identification of physical mixing based on concentrations of dissolved He and V, and the fraction of eDNA contributed by Archaea specifically adapted to deep groundwater conditions. Analytical data are summarized in Table 1 (the full dataset is provided as Supplementary Data 1 and 2).

**Table 1:** Overview of dissolved He, V and archaeal eDNA signatures of sampled springs and groundwater

Sample	ID	He (×10^−^^8^ cm^3^_STP_ g H_2_O^−1^)	^3^He/^4^He (×10^−6^)	^20^Ne/^4^He	He source			V (μg l^−1^)	*f*_A,YLA114_ (%)	*f*_A,WCHD3-30_ (%)	*H*′_A_
					Atm	Mnt	Rad				
Southwestern sub-basin											
Aoki	A	7.1	3.01	2.829	75%	20%	5%	221.0	49.4	45.8	1.27
Shibakawa no. 1	1a	3.7	1.71^53^	3.704^53^	100%			29.0	1.0^35^	19.9^35^	1.56^35^
Shibakawa no. 2	1b	4.6^53^	1.47^96^	3.765*	100%						
Jimbanotaki	2	3.8						16.3	12.4	40.9	1.79
Shiraitonotaki no. 1	5a	3.8^53^	1.86^53^	3.333^53^	90%	5%	5%	38.8			
Shiraitonotaki no. 2	5b	5.1^96^	1.79^96^	4.208*	100%						
Sugita	16	4.2						13.9			
Fujinishiki	F1							59.4	27.5	49.5	1.56
Yoshimaike	9	7.4	3.46	2.773	75%	20%	5%	87.5	35.0	45.3	1.41
Wakutamaike	10	149^96^	1.31^96^	0.139*	5%	12.5%	82.5%	46.5			
Southeastern sub-basin											
Mishuku	63	4.6						21.9			
Tomizawa	60	4.8	1.79	3.704	100%			30.4	22.0	55.0	1.39
Kakitagawa no. 1	48a	5.2	1.54	3.668	100%			37.5	23.3	13.9	1.64
Kakitagawa no. 2	48b	4.6^102^	1.68^102^	3.930^102^	100%						

Vanadium and archaeal eDNA signatures represent site-averaged values (full dataset provided as Supplementary Data 1). The three potential He sources that can mix to produce the observed ^3^He and ^4^He concentrations are the atmosphere (Atm), mantle (Mnt) or radiogenic decay (Rad)^54–57^. Note that in the context of groundwater hydrology, Neon isotopes are only of atmospheric origin^55^. STP stands for standard temperature and pressure (*T* = 0 °C, *P* = 1 atm). *f*_A,YLA114_ and *f*_A,WCHD3-30_ are the fractions of archaeal eDNA contributed by the candidate extremophile Parvarchaea orders YLA114 and WCHD3-30, respectively. *H*′_A_ is Shannon’s diversity index (*H*′)^160^ evaluated for Archaea. * ^20^Ne/^22^Ne ratios for these samples were not provided by Asai and Koshimizu^96^ and the ratio of air-saturated water was used instead. He concentrations from this study correspond to concentrations measured on site with the new GE-MIMS instrument.

Data from refs. 35, 53, 96, 102.

### Helium

Near volcanoes and plate borders, total He concentrations and the isotope ratios of ^20^Ne/^4^He and ^3^He/^4^He are important tracers, as they allow the contributions of atmospheric versus terrigenic He to be quantified, as well as the separation between mantle and radiogenic He^54–64^. For example, if in a volcanic and tectonically active system like Fuji catchment deep groundwater was to be found enriched in mantle He, this He signature can be used to detect the contributions of deep groundwater to shallow groundwater and constrain the origin of water at the sampled freshwater springs.

**Fig. 3:**
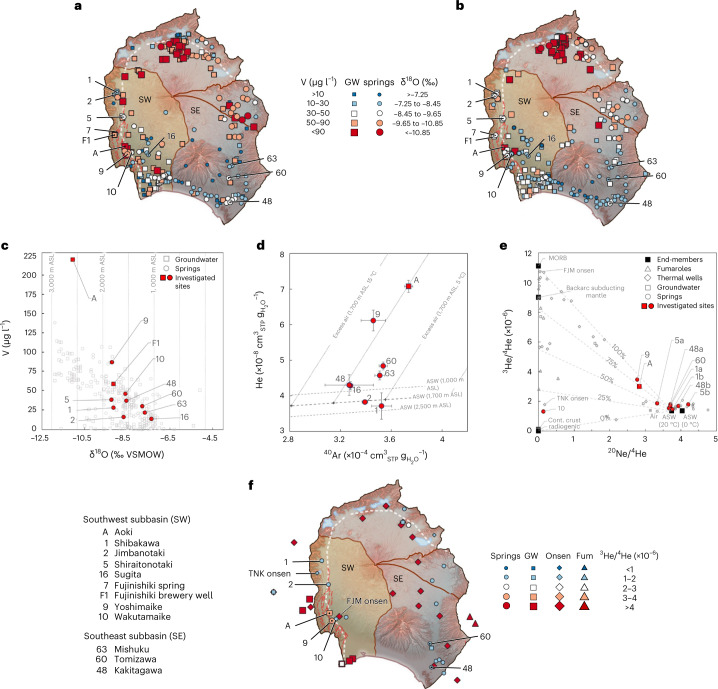
Overview of V, δ^18^O and He compositions in springs and groundwater of Fuji catchment. **a**,**b**, Maps of V (**a**) and δ^18^O (**b**) compositions. **c**, δ^18^O versus V concentrations. Hypothetical recharge elevations (from δ^18^O after Yasuhara et al.^36^) are indicated by dotted vertical lines assuming no mixing of waters from different elevations. **d**, Average He and ^40^Ar concentrations measured on site with the new portable GE-MIMS instrument^133^. The black dashed lines represent air-saturated water (ASW) for 20 °C at the three primary recharge elevations after Yasuhara et al.^36^ for the southwestern basin. The dotted lines indicate hypothetical excess air additions to ASW at 1,700 m ASL and for 0 °C, 5 °C, 10 °C and 15 °C. Error bars indicate ±1 s.d. of the mean of all GE-MIMS measurements taken at each site. The numbers of sampling dates used to quantify average values and standard deviations per site are: A (*n* = 2), 1 (*n* = 1), 2 (*n* = 1), 9 (*n* = 2), 16 (*n* = 1), 48 (*n* = 2), 60 (*n* = 2), 63 (*n* = 1). GW, groundwater. **e**, ^3^He/^4^He versus ^20^Ne/^4^He for samples obtained in this study (sites A, 6, 9 and 10a) alongside previous measurements (sites 1a^96^, 1b^53^, 3a^96^, 3b^53^, 7^96^ and 10b^102^ and data from onsen deep wells^53,104,107,111^, groundwater wells^53^ and fumaroles^57^) and end-member isotopic ratios^55,57^ (mid-ocean ridge basalt (MORB), continental crust, radiogenic production, ASW at 0 °C and ASW at 20 °C). **f**, Overview map of ^3^He/^4^He ratios. TNK, Lake Tanuki. FJM, Fujinomiya. Fum, Fumaroles. For all data and corresponding references, see Supplementary Data 1. Coordinate reference system: WGS 84 / Pseudo-Mercator. Background composite map sources: digital elevation model^162^; red 3D hillshade map^163,164^; active tectonic fault locations^165^; plate boundaries and major tectonic faults^43,166,167^. White dashed lines in **a**, **b** and **f** indicate the FKFZ and KMFZ tectonic zones; red dashed lines in **a**, **b** and **f** indicate active tectonic faults.

By taking the characteristic ^3^He/^4^He and ^20^Ne/^4^He ratios of air-saturated water (^3^He/^4^He = 1.36 × 10^−6^, ^20^Ne/^4^He = 3.741)^65^, depleted mantle (as sampled by mid-ocean-ridge basalt; ^3^He/^4^He = 1.1 × 10^−5^, ^20^Ne/^4^He ≈ 0)^55^ and continental crust (^3^He/^4^He = 1.5 × 10^−8^, ^20^Ne/^4^He ≈ 0)^57^ into account, the contribution of mantle He can be calculated: 20% in the Ko-Fuji deep groundwater of Aoki well and Yoshimaike spring, 12% in Wakutamaike spring, and 5% in Shiraitonotaki (sample no. 1) (Table 1 and Fig. 3e,f). The high mantle He contributions in Aoki and Yoshimaike correlate with high total He concentrations (Table 1 and Fig. 3d,e). Hence, noble gas data suggest that Ko-Fuji deep groundwater is contributing significantly to Fuji’s southwestern springs—less so at the northern, upstream end of the alluvial fans (Shibakawa, Jimbanotaki, Shiraitonotaki), but strongly in springs located directly on the FKFZ (Yoshimaike, Wakutamaike) (Figs. 1 and 3). Although the presence of mantle He in the different springs could be a result of the upwelling of Ko-Fuji deep groundwater, it could also be the result of a direct admixture of mantle gases. Additional tracers are therefore required to confirm the upwelling of Ko-Fuji deep groundwater.

The Wakutamaike spring exhibited not only a 12% contribution of mantle He, but also an 83% contribution of radiogenic He (Fig. 3e,f)—an observation that suggests a hydraulic connection to a completely different groundwater reservoir. Total He concentrations are also three orders of magnitudes larger than in any other sampled spring. The He signature is very similar to the signature of the deep thermal water pumped close to Lake Tanuki at the base of the Misaka-Tenshu Mountain range (Fig. 3e,f and Supplementary Section 3), suggesting that the complex network of faults, fissures and clinkers of the FKFZ transports groundwater from deep below the Misaka-Tenshu mountains to Wakutamaike spring with little mixing.

### Vanadium

Dissolved V has been measured in Fuji’s springs, groundwaters and rivers since the 1960s and is found to be enriched and prevail in oxidized form as vanadate (V(v)). Owing to the naturally high V content of basalt^66^, the V concentration in Fuji’s groundwater and springs is much larger than anywhere else in Japan^16,17,67–69^, making Fuji’s rivers the most V-enriched on Earth^15^. While V reaction kinetics in natural waters are difficult to quantify due to vanadium’s highly complex geochemistry^70–74^, on the scale of the decadal residence times of Fuji groundwater, equilibria concentrations are probably not reached^72,75^ and V concentrations can be assumed to increase gradually with groundwater residence time. This assumption is supported by the observation that V concentrations have not reached a plateau (Fig. 3c). Thus, if a spring was to be found to be significantly enriched in V compared to the local shallow groundwaters, upwelling of deep groundwater with its significantly longer residence times is the most likely cause of enrichment.

Vanadium concentrations exhibit similar patterns to stable water isotopes and major ions, with δ^18^O and V concentrations correlating almost perfectly and lighter waters, which are recharged at higher elevations, showing larger V concentrations (Table 1, Fig. 3a–c and Supplementary Section 2). Springs overall contain less V and are isotopically heavier than groundwater (Fig. 3a–c), confirming that most springs are fed by shallow groundwater from the Surficial and Shin-Fuji aquifers, which are generally characterized by low recharge elevations and short residence times^14,17,37^. Vanadium concentrations also positively correlate with He concentrations; that is, the He-rich waters tend to be enriched in V. With a concentration of 221.0 μg l^−1^, the Ko-Fuji deep groundwater sampled in Aoki well is the most V-enriched water found around Fuji, supporting the assumption of the very long residence time of Ko-Fuji groundwater in the southwestern foot of Fuji. Remarkably, with a concentration of 87.5 μg l^−1^, Yoshimaike spring contains significantly more V than all other springs in the southwestern sub-basin. Although the correlation between elevated V and elevated He concentrations in springs makes a strong case for substantial Ko-Fuji deep groundwater upwelling, elevated V concentrations in springs may also arise from increased residence times due to longer flow paths or zones of reduced hydraulic conductivity within the shallow aquifers themselves. Hence, another independent tracer is required to fully confirm the existence of substantial upwelling of deep groundwater.

### Microbial eDNA

Pioneering the investigation of microbial eDNA in waters around Fuji, Segawa et al.^34^ identified a potential relationship between the presence of thermophilic prokaryotes and deep groundwater flow paths through Ko-Fuji aquifer in Fuji’s southwestern sub-basin. Later, Sugiyama et al.^35^ confirmed that candidate extremophilic prokaryotes are cornerstone organisms in Ko-Fuji aquifer, and observed that a typhoon-induced torrential rainfall event resulted in substantially increased concentrations of suspended Archaea in Aoki well. Revisiting the eDNA data of Sugiyama et al.^35^, we found members of Parvarchaea to be the dominant Archaea in Fuji groundwaters; specifically, the two uncultivated candidate orders YLA114 and WCHD3-30, which are primarily retrieved from extreme environments^76,77^ (Table 1; a detailed phylogeny is provided in Supplementary Section 4). Although the total number of Archaea in suspension increased during the typhoon-induced torrential rainfall event, the relative contributions of WCHD3-30 and YLA114 decreased, indicating that WCHD3-30 and YLA114 are primarily living in suspension rather than attached to the aquifer matrix, and that their relative contributions are reduced when increased hydraulic gradients lead to an increased detachment of Archaea that live attached on the matrix under normal hydraulic conditions. This property makes both WCHD3-30 and YLA114 potential tracers of upwelling of deep groundwater into shallow groundwater and springs in Fuji catchment even under normal hydraulic conditions. As the environmental conditions that allow these specific Archaea to develop have so far been found around Fuji only at great depths^17,52,78,79^, the presence of the DNA of these microbial life forms in springs is furthermore likely to be indicative of fast upwelling of a significant amount of deep Ko-Fuji groundwater, as these specific DNA would otherwise not exist or be degraded (in the case of marginal or slow deep groundwater upwelling).

To confirm the previously identified link between the presence of these specific prokaryotes and the environmental conditions prevalent in Ko-Fuji deep groundwater^34,35^, and to allow comparison between dissolved He, V and eDNA, we also determined the microbial eDNA present in water samples of the investigated springs and Aoki well. This revealed that Parvarchaea account for 95% of all archaeal eDNA present in the Ko-Fuji groundwater of Aoki well (Table 1 and Supplementary Section 4). While in the most upstream of the sampled southwestern springs (Shibakawa), Parvarchaea account for only 20% of archaeal eDNA, this percentage increases gradually in the downstream direction and reaches 80% in Yoshimaike spring. Parvarchaea are also the most important Archaea in Tomizawa (77%), an upstream southeastern spring emerging at the foot of Mt Ashitaka, and also present in significant levels (albeit not as dominant) in Kakitagawa (37%), the largest and most downstream spring of the southeastern sub-basin (Fig. 1). The clear spatial distribution of these Parvarchaea (that is, their comparably high abundance in downstream springs in the southwestern and southeastern sub-basins) thus makes a strong case for spatially increasing upwelling of Ko-Fuji deep groundwater in the downstream direction, particularly along the FKFZ. However, despite archaeal eDNA patterns agreeing with patterns in He and V concentrations, only a systematic comparison between these three different and completely independent tracers would allow the possibility of those Parvarchaea growing locally in springs in significant levels to be excluded and provide proof that Ko-Fuji deep groundwater is indeed upwelling.

## Evidence for mixing of groundwaters from different depths

**Fig. 4:**
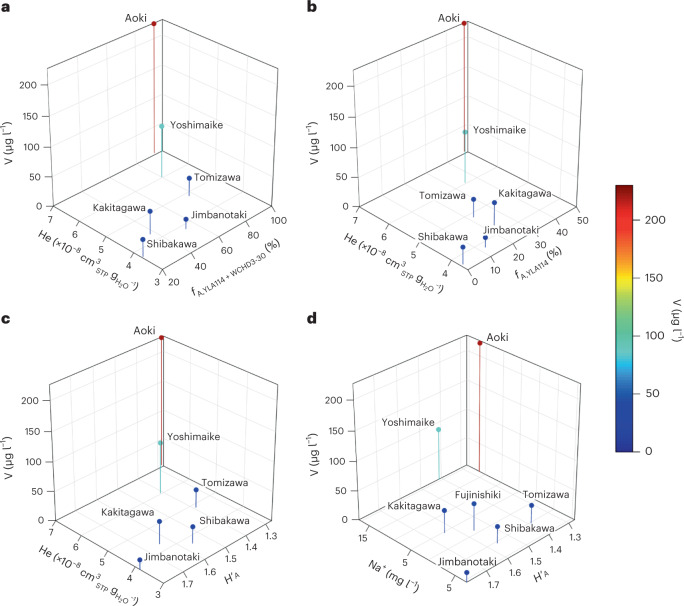
Triple tracer correlations between V, He and eDNA in Fuji catchment. **a**–**b**, Triple tracer correlations between V, He and archaeal eDNA contributed by YLA114 + WCHD3-30 (**a**) and by YLA114 only (**b**). **c,** Triple tracer correlation between V, He and the alpha diversity of Archaea. **d**, Triple tracer correlation between V, Na^+^ and the alpha diversity of Archaea (**d**). He concentrations represent measurements taken on site with the new portable GE-MIMS instrument. STP, standard temperature and pressure (*T* = 0 °C, *P* = 1 atm). *H*′_A_ is Shannon’s diversity index^160,168^ evaluated for Archaea. Data for Fujinishiki in **d** represent data from Fujinishiki brewery well (site id: F1), except for the Na^+^ concentration, which represents the average measured Na^+^ concentration in Fujinishiki spring (site id: 7) located next to the Fujinishiki brewery well.

To identify whether the three independent tracers indicate substantial upwelling of Ko-Fuji deep groundwater into the springs along the FKFZ, they are directly compared in four triple tracer plots (Fig. 4). Comparing the concentrations of dissolved V and He to the fractions of archaeal eDNA contributed by YLA114 (Fig. 4a) and by YLA114 + WCHD3-30 (Fig. 4b) reveals that the three tracers are nearly linearly correlated. The near-linear correlation prevails if concentrations of V and He are compared with the alpha diversity of Archaea (Fig. 4c). As all three tracer types have completely different biogeochemical origins, the only plausible explanation for the near-linear correlation, given current knowledge of the system, is physical mixing processes (upwelling and increasing admixture in the downstream direction) of Ko-Fuji deep groundwater into the shallow aquifers and freshwater springs of Fuji.

**Fig. 5:**
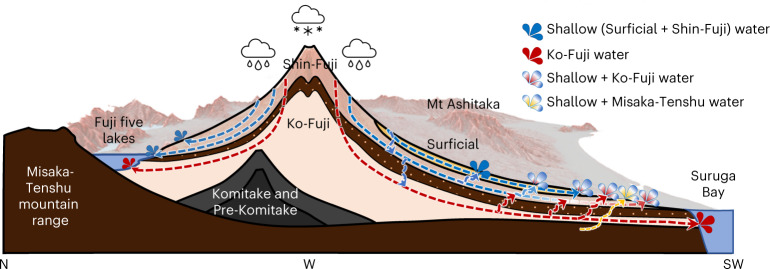
Revised conceptual hydrogeological model of Mt Fuji. The revised conceptual hydrogeological model of Mt Fuji (revised based on previously published conceptual models^7,17,36,42,169^) shows a north–southwest cross-section that follows the FKFZ and illustrates the prevailing vertical interactions between the three different principal aquifers (Surficial, Shin-Fuji and Ko-Fuji) and the resulting spring water origins. The contribution of Misaka-Tenshu deep groundwater to the sacred Wakutamaike spring is also illustrated. Blue arrows indicate shallow groundwater, red arrows Ko-Fuji groundwater and yellow arrows Misaka-Tenshu groundwater flow. Background composite map sources: digital elevation model^162^; red 3D hillshade map^163,164^.

The identified triple tracer correlation between He, V and extremophile archaeal eDNA, in combination with the near-linear correlations between V and δ^18^O, He concentrations and ^3^He/^4^He ratios and the relative abundance of the YLA114 and WCHD3-30 Archaea orders, provide strikingly strong evidence for widespread upwelling and admixture of Ko-Fuji deep groundwater into the springs and shallow aquifers of Fuji. The rate of upwelling of deep groundwater is far greater than previously assumed, particularly in the most densely populated southwestern sub-basin. The currently accepted hydrogeological model, which postulates negligible to no vertical mixing between the different groundwater bodies, is incompatible with the new tracer data and therefore needs to be revised. We propose a revised conceptual hydrological flow model of Fuji that explicitly assumes substantial upwelling of Ko-Fuji deep groundwater along the faults, fissures and clinkers of the FKFZ, which are a result of the complex subduction dynamics of Fuji triple junction (Fig. 5). In addition to these interaction pathways, we identified a previously unknown admixture of heavy He-enriched Misaka-Tenshu-type deep groundwater in Wakutamaike spring. Wakutamaike spring, coincidentially, is the sacred spring of Fujisan Hongu Sengen Taisha shrine, UNESCO World Heritage Site and one of Japan’s most important shrines.

## Conclusions

While groundwater level observations and classic hydrological tracers such as major ions and stable water isotope compositions of groundwater and spring water can provide valuable insights into hydrogeological systems, they cannot detect the vertical exchange between the different aquifers of Mt Fuji. Although applied widely, they are of limited use in many complex environments, as classic tracers are often confounded by interactions with the aquifer matrix, which typically consists of largely the same material and thus harmonizes the chemical composition of different waters, or by recharge zones that overlap, which harmonize the isotope compositions of different waters. By combining multiple classic and unconventional tracers—namely on-site analysis of dissolved (noble) gases, laboratory-based analysis of noble gas isotopes, next-generation sequencing of microbial eDNA, and the analysis of trace elements, all of which are tracers that specifically react to the variety in flow paths and processes that can be expected in a volcanic system such as Fuji catchment, and allow us to disentangle and therefore track waters that are subject to physical mixing—we not only overcame the principal limitations of the classic methods, but also demonstrated a clear way forward for groundwater science.

In conclusion, advances in analytical techniques in tracer hydrology and microbiology enabled us to understand the complex hydrogeology of the volcanic groundwater system of Fuji and to identify previously unknown upwelling of deep groundwater into freshwater springs and shallow groundwater. The combination of He, V and microbial eDNA signatures thus not only broadens the hydrogeological understanding of Fuji, but also showcases the vast potential of combining unconventional tracers to study complex hydrogeological systems.

## Methods

### Study site and current challenges

As a result of its location directly on top of the triple junction between the Okhotsk, Amur and Philippine Sea plates, Mt Fuji’s ejecta consist primarily of high-alumina basalt and volcanic ash, as opposed to the andesitic composition that most other stratovolcanoes located on the Izu–Bonin–Mariana arc eject^6^. The basaltic composition provides evidence that Fuji’s magma reservoir is located at a great depth (>20 km)^6,11,12,80–84^. Fuji consists of four volcanoes that grew on top of each other: Pre-Komitake (270–160 ka), Komitake (160–100 ka), Ko-Fuji (100–10 ka) and Shin-Fuji (10 ka to present)^1,6,38,81,85,86^. The deposits of the late Hoshiyama volcanic stage (100–17 ka) and deposits of the Fujinomiya and Subashiri stages (<17 ka)^9,13,17,31,35,37,39,87–89^ are of hydrogeological relevance. Late Hoshiyama deposits consist of basaltic lava, volcanic ash and respective mudflows, and host the deep Ko-Fuji aquifer. Ko-Fuji aquifer is confined on top by largely impermeable mudflow deposits (hydraulic conductivities between 10^−6^ m s^−1^ (horizontal) and 10^−8^ m s^−1^ (vertical); ref. 42), pyroclastic rocks and Fuji black soil of the final Hoshiyama and initial Fujinomiya stages^6,9,17,37,38,81,85,90^. The estimated hydraulic conductivity of Ko-Fuji aquifer is in the range of 10^−5^–10^−7^ m s^−1^ (refs. 9, 39, 42, 91). The Funjinomiya and Subashiri stage deposits host the shallow Shin-Fuji aquifer, which consist of multiple basaltic lava layers that form a complex and highly conductive network of porous material, fissures and clinkers^7,17,31,37^. The most recent volcanic ash and alluvial sand and gravel deposits of the Subashiri stage finish off the hydrogeological stratigraphy by hosting the uppermost Surficial aquifer^31^. The estimated hydraulic conductivities of the Shin-Fuji and Surficial aquifers are 10^−2^−10^−5^ m s^−1^ (refs. 9, 39, 42, 91). Underneath, the described hydrogeological system of Fuji is constrained by an approximately 10-km thick basement body of the Misaka-Tenshu group, which consists of impermeable submarine basaltic andesite and pyroclastic material^52^. The FKFZ, Japan’s tectonically most active structure, is located along the west and southwest foot of Fuji and passes the city of Fujinomiya^10,43–45^. These active tectonic faults are characterized by complex fissure and clinker networks, which might allow groundwater, solutes and small particles to be transported in a non-laminar fashion and make their flow paths very difficult to identify. Hydrogeological properties of the FKFZ, as well as its effect on groundwater dynamics and flow paths, have not been systematically investigated and, while geologically relatively well understood^45^, its hydrogeological behaviour remained unknown before our study.

At Fuji’s summit, the mean annual air temperature is −6.4 °C, and the mean temperature during the warmest month (August) is 6.0 °C (ref. 92). Annual rainfall ranges from 1,600–2,000 mm, depending on the orientation^9,93^. Snowfall occurs throughout the year and amounts to an annual total of 3 m at the summit^92^. These winter hydrological conditions sustain a layer of permafrost near the summit, effectively preventing any infiltration into the subsurface^36,94^. However, conditions are slowly changing as a result of climate change (for example, mean maximum temperatures during the summer months have risen by 2 °C during the past 50 yr and the timberline is climbing^95^). Of the 2.2 km^3^ of precipitation that fall on Fuji each year, 2 km^3^ infiltrate and form groundwater that feeds Fuji’s three aquifers (the Surficial, Shin-Fuji and Ko-Fuji aquifers). Groundwater then flows down Fuji’s flanks and, after 2–4 decades^14,96^, 1.7 km^3^ emerge again each year in the foothills, feeding countless springs, rivers and lakes^37,39,49,52^. The remaining 0.3 km^3^ leave the catchment as regional groundwater (for example, towards Katsura River Valley in the north) or as submarine groundwater discharge (to Suruga Bay to the south^33^).

According to stable water isotopes, groundwater recharge on Fuji occurs at three different elevations: above 2,000 m ASL (upper zone), between 1,100 and 2,000 m ASL (intermediate zone) and below 1,100 m ASL (spring zone)^9,17,36,50^. Recharge in the southwestern sub-basin occurs primarily between 1,600 and 2,250 m ASL, with the bulk of recharge taking place in the intermediate zone and feeding Shin-Fuji aquifer^36^. While the upper zone is of little importance for bulk groundwater recharge, it is the principal zone for recharge of Ko-Fuji aquifer^36^. The spring zone is characterized by (1) the emergence of a large amount of groundwater into the countless freshwater springs located along the end of the Shin-Fuji lava formation, (2) dynamic exchanges between springs, streams, rivers and shallow groundwater, and (3) agricultural, urban and industrial water use. In contrast to many regions around Japan, Fuji’s natural springs are exclusively cold-water springs, and the spas that advertise as "hot springs" (*onsen*) around Fuji pump their water from Fuji’s basement at a relatively uniform depth of 1,500 m.

Fuji is also known as the water mountain in Japan and is well known for its pristine and abundant springs and groundwater. Owing to its long residence time in basaltic rocks, Fuji’s springs and groundwater are very soft (that is, devoid of carbonates) and naturally enriched in V, making the water important for green tea cultivation and mineral health water, whiskey and sake production^15–17,22,23,25–28,97^. Fuji’s water quality and abundance, however, have been in steady decline throughout the past few decades, resulting in the region around Fuji not receiving the originally envisaged UNESCO World Natural Heritage Site designation and instead ‘only’ a UNESCO World Cultural Heritage Site denomination, as the environmental requirements for the former were too strict^29^. The decline in Fuji’s water quality and quantity is mainly related to the steady decline in lake and groundwater levels due to over-pumping, widespread groundwater pollution due to industry and paper production, excessive nutrient inputs (for example, nitrate) from green tea cultivation, increasing water temperatures and changes in the hydrological cycle due to climate change, and illegal waste dumping^1,18,29–31,39,98^. In this context, the most impacted region is the urbanized area surrounding Fujinomiya, which is affected by industry, large green tea plantations in the uphill slopes, and the FKFZ (Fig. 1), within which the groundwater dynamics are only vaguely understood^17,34,35,99^. Because the conceptual notion of purely laminar groundwater flow persisted and led to an inability to close the local water balance of Fuji catchment, important groundwater pathways and fluxes remained unidentified^29,30^. Understanding the pathways and associated flow fields, however, is a precondition for preventing and managing contamination of groundwater and springs.

### Investigated sites and existing data

While we present data for the entire Fuji catchment, our focus was on identifying the origins of the water in the freshwater springs along the southwestern foot of Fuji, as it is that region that is most affected by both agriculture and industry, while at the same time being the most complex hydrogeologically due to the FKFZ. The investigated springs and artesian groundwater wells are hydrologically important features and are all located along the principal groundwater flow directions in the southwestern sub-basin (flow direction: Shibakawa spring, Sugita spring, Jimbanotaki spring, Shiraitonotaki spring, Fujinishiki sake brewery artesian well, Aoki artesian well, Yoshimaike spring, Wakutamaike spring; Fig. 1). Many of the sites sit directly on top of the FKFZ, where groundwater dynamics between the different aquifers, springs and surface water bodies are expected to be highly complex. For example, in response to the *M*_w_ 5.9 East Shizuoka earthquake in March 2011, which itself was triggered by the major *M*_w_ 9 Tohoku Oki earthquake, several springs and groundwater wells overflowed^30,43,44,100,101^. To complete a regional understanding of groundwater dynamics, three important springs of the southeastern sub-basin were also investigated (flow direction: Mishuku spring, Tomizawa spring, Kakitagawa spring; Fig. 1). Table 2 lists the different tracer analyses available for these sites and includes our measurements, as well as older data available from the literature.

**Table 2:** Overview of investigated springs and artesian groundwater wells

Site	Id	Latitude (° N)	Longitude (° E)	Elevation (m ASL)		Well depth (m)	Type	Major ions	δ^2^H & δ^18^O	Residence time	^87^Sr/^86^Sr	V	NG	TDC	eDNA
Shibakawa	1	35.3717	138.5672	715			spring	x,o	x,o	o	o	o	x,o	x,o	o
Jimbanotaki	2	35.3663	138.5612	695			spring	x,o	x,o	o	o	o	x	x,o	x
Shiraitonotaki	5	35.3128	138.5876	480			spring	o	o	o	o	o	o		
Fujinishiki	7	35.2692	138.5597	230			spring	o	o	o	o	o			
	F1	35.2692	138.5597	230	32		well		x			x		x	x
Yoshimaike	9	35.2231	138.5982	130			spring	x,o	x,o	o	o	x,o	x	x,o	x
Wakutamaike	10	35.2276	138.6108	120			spring	o	o	o	o	o	o	x,o	
Aoki	A	35.2420	138.5910	140	550		well	x,o	o			x	x	x,o	x
Sugita	16	35.2221	138.6600	195			spring	o	x,o	o	o	o	x	x,o	
Mishuku	63	35.1961	138.9059	175			spring	o	x,o	o		o	x	x,o	
Tomizawa	60	35.1662	138.8956	110			spring	x,o	x,o	o		o	x	x,o	x
Kakitagawa	48	35.1077	138.9003	15			spring	x,o	x,o	o	o	x,o	x,o	x,o	x

NG, noble gases; TDC, microbial total direct cell counts. Data for Fujinishiki are composed of data from the Fujinishiki sake brewery’s artesian well (site id: F1; all data except for major ions and Sr isotope ratios) and Fujinishiki spring (site id: 7) located next to the well.

x, this study; o, existing literature.

We compiled our measurements and the available literature data into a hydrogeological dataset on Fuji catchment, encompassing more than 350 sites and over 9,500 individual data points^7,14,17,31,34,35,37,41,48,52,53,57,87,88,96,97,99,102–116^. All sites for which hydrochemical data were available that we addressed here are marked in Fig. 1. The complete dataset is provided as Supplementary Data 1, except for the microbial eDNA-based phylogenetic data, which are provided as Supplementary Data 2.

### Major ions and stable water isotope analyses

For the analysis of stable water isotopes and major ions, samples were filtered with a 0.22 μm Millex-GS filter (Merck Millipore) and stored at −20 °C and 4 °C, respectively, before analysis. Major ion compositions were analysed at Shizuoka University using a Dionex ICS-3000 ion chromatograph (Thermo Fisher). Stable water isotopes were analysed by Shoko Science Co Ltd (using a Picarro L2120-*I* cavity ring-down spectrometer, Picarro, Inc.), normalized to the VSMOW and reported in δ notation^117^ (typical analytical errors are ±0.2‰ for δ^2^H and ±0.05‰ for δ^18^O).

### V and Sr isotope analyses

Owing to their low abundance and very diverse concentrations and isotopic ratios in different rocks and minerals, V concentrations and Sr isotopes are powerful geochemical tracers of groundwater flow and proxies of groundwater residence times^118,119^. Vanadium in groundwater originates from alkaline rocks in contact with oxidized water, and is found to increase with groundwater residence times^16,70,71,120^. Vanadium dissolution is geochemically similar to Sr enrichment in groundwater. In groundwater hydrology, the ^87^Sr/^86^Sr isotopic ratio is widely employed as a tracer to track exchanges with different rocks and minerals^119,121–124^. As water–rock exchange processes depend on time, V concentrations and ^87^Sr/^86^Sr ratios tend to correlate with, and (under certain constraints) might be indicative of, groundwater residence times^46,51,125^.

Vanadium water samples were filtered using a 0.22 μm Millex-GS filter (Merck Millipore) and acidified to a pH below 2 using nitric acid. The filters were washed with 10% hydrochloric acid and 0.1 M nitric acid before use. Vanadium concentrations were analysed using a polarized Zeeman Z-3700 atomic absorption spectrophotometer (Hitachi High-Tech) at Shizuoka University after dilution with 0.1 M nitric acid by 10% (typical analytical error ±4%). Strontium isotopic ratios were taken exclusively from the literature.

### Dissolved (noble) gas analyses

Concentrations of dissolved noble gases and their isotopic ratios have been employed as groundwater tracers in many hydrogeological contexts, ranging from palaeotemperature reconstruction, excess air quantification, recharge elevation identification, and the quantification of the mixing of waters of different origins^55,126–132^.

On-site dissolved (noble) gas analyses were carried out using gas equilibrium-membrane inlet mass spectrometry (GE-MIMS)^133^. GE-MIMS allows simultaneous measurement of inert and reactive gases (He, ^40^Ar, ^84^Kr, N_2_, O_2_, CO_2_, H_2_ and CH_4_) in air and dissolved in water directly on-site, in near real time (complete analysis ~15 min) and with typical analytical uncertainty of ±1–3% (ref. 133). For the dissolved gas analyses, groundwater was pumped through a flow-through membrane contactor (G542 Liqui-Cel MiniModule, 3 M) at approximately 2 l min^−1^ using a peristaltic pump. The extracted gases were subsequently transferred via a 10 m stainless steel capillary to a quadrupole mass spectrometer (RGA 200, Stanford Research Systems) for final detection. For ^40^Ar, N_2_, O_2_ and CO_2_, which are more abundant, each data point represents the average of five individual measurements taken over a roughly 2-min period, with standard errors being approximated by the standard deviation of these five measurements. For the comparably less abundant gases ^4^He, ^84^Kr and CH_4_, each data point represents the average of 15 individual measurements taken over an approximately 4-min period, with standard errors being approximated by the standard deviation of these 15 measurements. For more experimental detail, refer to refs. 133, 134. Mass spectrometric data were processed using the ruediPy package (v2019)^135^ in Python (v3.8)^136^.

High-resolution noble gas isotope analyses were carried out on samples of approximately 25 g of water collected in copper tubes^126^ at the Swiss Federal Institute of Technology Zurich following standard protocols (see ref. 137; typical analytical errors are <1% for He and Ne concentrations, and <0.5% for isotopic ratios). As the standard protocol by Beyerle et al.^137^ employs the standard atmospheric ratio of ^3^He/^4^He (*R*_a_) of 1.384 × 10^-6^ as determined by Clarke et al.^138^, the few literature-based dissolved ^3^He/^4^He ratios (*R*) reported in the *R*/*R*_a_ format were converted to the ^3^He/^4^He format on the basis of this ratio.

### Microbial eDNA analyses

Microorganisms have been used intensively to study biogeochemical processes in surface water and groundwater, but only few studies have used microorganisms to study physical processes such as groundwater flow paths or groundwater mixing^139–142^. Recently, however, conceptual understanding of the movement of microorganisms in groundwater has improved, and it is now more widely acknowledged that microorganisms can travel over considerable distances^35,143,144^ and can survive for years^145^. These aspects make microorganisms promising tracers of groundwater flow over relevant spatial and temporal scales. Furthermore, next-generation sequencing now allows the phylogenetic composition and functions of microbial communities to be identified on the basis of the analysis of microbial eDNA present in water samples in an affordable, quantitative and highly efficient manner^146^.

Water samples for the analysis of microbial eDNA were collected in this study by filtering 10 l of water using 0.22 μm Sterivex-GV filters (EMD Millipore). DNA extraction was carried out at Shizuoka University using standard protocols^147^, whereby prokaryotic cells were first lysed from the 0.22 μm Sterivex-GV filter units by adding a solution of lysozyme and proteinase K. Bulk DNA was then extracted using a phenol–chloroform–isoamyl alcohol mixture^148^ and subsequently quantitatively determined by spectrophotometry using a NanoVue spectrophotometer (GE Healthcare UK Ltd). Amplification and sequencing were carried out by Bioengineering Lab Co. Ltd. In a two-step PCR, the hypervariable V3–V4 regions of the bacterial and archaeal 16S rRNA gene were amplified using the universal 341F/805R primer pair. In the first PCR step, the 1st-341f_MIX (5′-ACACTCTTTCCCTACACGACGCTCTTCCGATCT-NNNNN-CCTACGGGNGGCWGCAG-3′)/1st-805r_MIX (5′-GTGACTGGAGTTCAGACGTGTGCTCTTCCGATCT-NNNNN-GACTACHVGGGTATCTAATCC-3′) primer pair was used and, after purification of the PCR products, in a second PCR step the 2ndF (5′-AATGATACGGCGACCACCGAGATCTACAC-ACACTCTTTCCCTACACGACGC-3′)/2ndR (5′-CAAGCAGAAGACGGCATACGAGAT- GTGACTGGAGTTCAGACGTGTG-3′) primer pair was used^149–151^. Sample libraries for next-generation sequencing with MiSeq (Illumina Inc.) were prepared using the MiSeq Reagent Kit v3 (Illumina Inc.), following manufacturer protocols. Amplicon sequencing was done via paired-end sequencing (2 × 300 bp) on the MiSeq platform. Operational taxonomic units were clustered at a 97% similarity level using QIIME 2^152,153^ and assigned on the basis of representative sequences by comparison against the GreenGenes database (v13.8)^154,155^. Patterns in the microbial community structure were explored using the phyloseq package (v1.30.00)^156^ in R (v3.6.2)^157^. The nucleotide sequence datasets obtained in this study have been filed in the DNA Databank of Japan (DDBJ) under accession number DRA013474.

### Microbial enumeration based on total direct counts

Total direct counts of microbial cells (a rapid method for the quantification of both culturable and unculturable microorganisms in environmental samples) were conducted at Shizuoka University following standard procedures^158^. A 100 ml water sample was collected on a 0.2 μm Nuclepor filter (GE Healthcare UK Ltd) and fixed with pH-neutral formaldehyde. The prokaryotic cells captured on the filter were then stained with 0.01 μg ml^−1^ fluorescent 4′,6-diamidino-2-phenylindole (Nacalai Tesque Inc.) and counted optically (using a BX51-FLA epifluorescence microscope equipped with a DP71 camera (Olympus)).

### Reporting summary

Further information on research design is available in the Nature Portfolio Reporting Summary linked to this article.

### Supplementary information

Supplementary Information Supplementary Figs. 1 and 2, Results and Discussion.

Reporting Summary

Supplementary Data 1 Mt Fuji hydrogeochemical dataset.

Supplementary Data 2 Mt Fuji spring and groundwater microbial eDNA dataset.

## Data Availability

All data used in this study are compiled into Supplementary Data 1 and 2 and are available via the public data repository HydroShare at 10.4211/hs.4eac370d12e142b5aa718e5deb57da39 (ref.^159^). The nucleotide sequence datasets obtained in this study are filed in the DNA Databank of Japan (DDBJ) under accession number DRA013474. Unless stated otherwise, hydrogeochemical and map background data were obtained with the open-source web browser Mozilla Firefox (v.68-v98), maps were generated with the open-source geographical information system QGIS (v3.6-v3.18) and data were processed with Microsoft Office (v2016-v2019) for Mac.
