# Supplementary material for: Revisiting Mt Fuji’s groundwater origins with helium, vanadium and environmental DNA tracers
Source: Nat Water. 2023 Jan 19;1(1):60–73. doi: 10.1038/s44221-022-00001-4 (PMC11041693; doi:10.1038/s44221-022-00001-4)
Supplement: Supplementary file 1 — Supplementary Figs. 1 and 2, Results and Discussion. [file 44221_2022_1_MOESM1_ESM.pdf]

nature water

Article https://doi.org/10.1038/s44221-022-00001-4

Revisiting Mt Fuji's groundwater origins with helium, vanadium and environmental DNA tracers

In the format provided by the authors and unedited

Table of contents

Section 1) Extended Results: Major ions and stable water isotopes

Section 2) Extended Results: Vanadium and $^{87}Sr/^{86}Sr$

Section 3) Extended Results: Helium and other dissolved noble gases

Section 4) Extended Results: Microbial eDNA

References

Extended results: Major ions and stable water isotopes

Our dataset covers the entire spectrum of groundwater and spring water stable water isotope and major ion composition present in Fuji catchment (see **Fig. 2**). Hydrochemical end members of the spectrum are represented by the Ko-Fuji deep groundwater sample from Aoki well (sampling depth = 550 m), and the Sugita spring water, which represents the very recent groundwater of the Surficial aquifer. While the deep Ko-Fuji groundwater is isotopically light and thus recharged at high elevations, and the most enriched in Na^+, the water of Sugita spring is isotopically heavy and thus recharged at low elevation, as well as contaminated by fertilizers used in green tea cultivation[1]. Most other springs show isotopic and major ion compositions that fall between these two end members.

As the stable water isotope signature in precipitation is a function of the geographical position where precipitation falls, δ^2H and $\delta^{18}O$ values of springs and groundwater have been successfully used to identify the approximate recharge elevations of the respective waters on the flanks of Fuji. Stable water isotopes revealed an elevational difference in recharge of the shallower Surficial and Shin-Fuji aquifers (generally between 1,100-2,000 m ASL) versus that of the deeper Ko-Fuji aquifer (generally above 1,500 m ASL)[2]. However, due to the shared meteoric origin of all groundwater in Fuji's flanks and alteration of the isotopic composition of water due to water-rock exchange and groundwater mixing, δ^2H and $\delta^{18}O$ results at Fuji were found to not be specific enough to rigorously assess groundwater flow paths or physical mixing between different groundwater types[3-7]. Moreover, with the exception of the impermeable basement, which consists of basaltic green tuff of the Middle Miocene Misaka and Tanazawa groups[8,9], Mt. Fuji is the product of volcanic activity on top of *Fuji triple junction,* the trench-trench-trench meeting point of the Okhotsk, Amur and Philippine Sea plates[10]. Consequently, the springs and groundwaters of both the shallow Surficial and Shin-Fuji aquifers as well as the deep Ko-Fuji aquifer develop and evolve in very similar geochemical conditions.

In conclusion, due to shared meteoric origins and similar aquifer material, both stable water isotopes and major ions vary in a way that could be readily explained by primarily laminar flow conditions. As primarily these two classic tracers were considered in the conceptual groundwater development for the Fuji hydrogeological system so far, and as the general absence of hot springs in Fuji catchment doesn't readily indicate widespread deep groundwater upwelling, potential vertical interactions between the three different aquifers remained masked and the conceptual view of Fuji being a simple, laminar groundwater flow system persisted.

Extended results: Vanadium and $^{87}Sr/^{86}Sr$

As indicated in **Fig. 3**, groundwater in the northern subbasin of Fuji is more enriched in vanadium and isotopically lighter than groundwater in the southwestern and the southeastern

subbasins. In line with previous findings, groundwater in the northern subbasin thus seems to have recharged at higher elevations compared to groundwater in the southern subbasins. The elevated vanadium concentrations in shallow groundwater and springs in the norther subbasins, however, are not indicating vertical interactions between the different aquifers but instead are a result of the fact that Shin-Fuji aquifer ends well before the emergence of most springs on the lower reaches of the northern Fuji subbasin, thereby making Ko-Fuji aquifer the only possible aquifer from which springs and groundwater can derive in this region[2,11,12].

In Fig. S1, the isotopic ratio of $^{87}Sr/^{86}Sr$ is illustrated alongside vanadium concentrations. In general, groundwater and springs enriched in vanadium also have lower $^{87}Sr/^{86}Sr$ ratios that closely correspond to the ratios of the Fuji aquifers' source materials (Shin Fuji ≈ 0.70344 & Ko-Fuji ≈ 0.70339 [13]; Komitake & Pre-Komitake ≈ 0.70337 [14]; backarc subducting crust ≈ 0.7033 [15]). Waters with low vanadium concentrations have $^{87}Sr/^{86}Sr$ ratios typical for the ratio in local precipitation (~0.709-0.71 [16]). These results indicate that $^{87}Sr/^{86}Sr$ ratios evolve quickly towards the background ratios of the aquifer source material: Once the groundwater residence time has been large enough to allow groundwater to accumulate 20 µg/L (for most samples) to 40 µg/L (for a small subset of the samples) of vanadium, $^{87}Sr/^{86}Sr$ ratios plateau close to the $^{87}Sr/^{86}Sr$ ratio of the Fuji aquifer source materials. Unfortunately, most existing $^{87}Sr/^{86}Sr$ data are not reported at high precision and produce an unnatural plateau at precisely 0.70375. This fact makes vanadium a better suited tracer to distinguish waters from the different Fuji aquifers compared to the low-precision $^{87}Sr/^{86}Sr$ ratios.

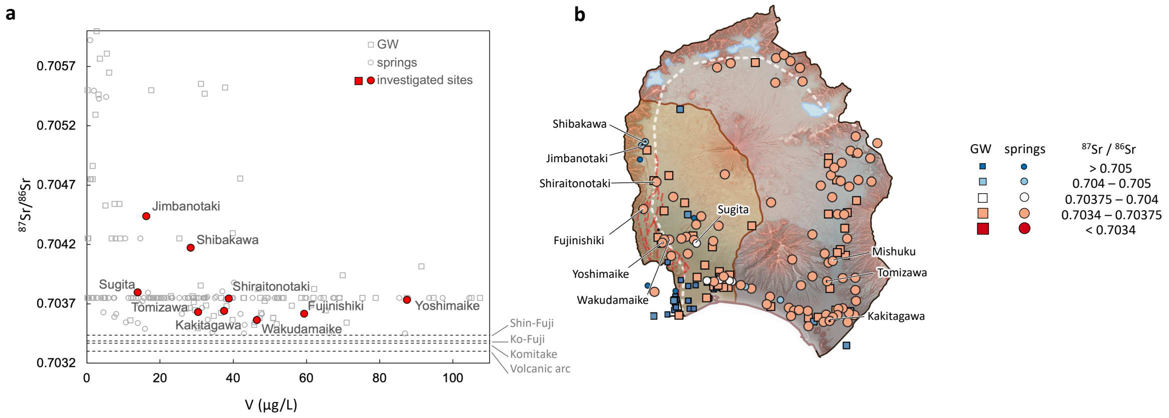

Figure S1: $^{87}Sr/^{86}Sr$ in springs and groundwater around Mt. Fuji. **a** Plot of $^{87}Sr/^{86}Sr$ vs. V; average $^{87}Sr/^{86}Sr$ ratios of Shin-Fuji (0.70344 [13]), Ko-Fuji (0.70339 [13]), Komitake (0.70337 [14]), and backarc subducting crust (0.7033 [15]) are indicated by horizontal dashed lines. **b** Overview map of $^{87}Sr/^{86}Sr$ ratios; key sites are labelled and locations for which microbial eDNA were carried out are indicated by black dots. All data and corresponding references are provided in tabular form in **Extended Data Table 1**. Background composite map sources: Digital elevation model[17]; red 3-D hillshade map[18,19]; Active tectonic fault locations[20]; Plate boundaries and major tectonic faults[21-23]. Coordinate reference system: WGS 84 / Pseudo-Mercator.

Extended results: Helium and other dissolved noble gases

With respect to the noble gas measurements illustrated in **Fig. 3d** and conducted on-site with the portable GE-MIMS technology[24,25], most springs have total He and ^{40}Ar concentrations close to those of air-saturated water for a characteristic range of temperatures (5-10°C) and recharge elevations (1,000 and 2,500 m ASL). The southeastern springs Tomizawa and Mishuku are slightly enriched in He relative to ^{40}Ar. Such an excess of light noble gases is typical for groundwaters which contain an excess in air resulting from air bubble entrapment and dissolution during recharge[26]. However, the Ko-Fuji deep groundwater of Aoki well and the Yoshimaike spring show significantly higher helium excess than all other springs. These significant surpluses can neither be explained by the addition of excess air, as the $^{3}He/^{4}He$ ratio is much larger than that of air (**Fig. 3e**), nor by tritiogenic production of ^{3}He, as the observed

^{3}He enrichment would require much larger ^{3}H inputs than have been observed in Japan[27], nor by *in situ* radiogenic ^{3}He and ^{4}He production. These findings, together with the observation of relatively short groundwater residence times (<100 years[11]), thus lead to the conclusion that the observed ^{3}He and ^{4}He enrichment in Ko-Fuji deep groundwater, as sampled in Aoki well, and in Yoshimaike spring water are the result of upwelling of terrigenic He. Such an injection of terrigenic He seems most likely to occur through the faults, fissures and clinkers of the FKFZ, either through admixture of gas or of even deeper groundwater enriched in terrigenic He to the Ko-Fuji deep groundwater and Yoshimaike spring.

The separation of atmospheric vs. terrigenic He as well as the determination of ^{3}He/^{4}He ratios of terrigenic He sources (e.g., mantle vs. crustal) is achieved by correlating the ^{3}He/^{4}He with the ^{20}Ne/^{4}He isotope ratios in a 3-isotopes-plot (see Kipfer, et al. [26] or Sano and Fischer [28]; **Fig. 3e**). Except for Yoshimaike, Wakutamaike and Shiraitontaki (sample #1), all springs analyzed in this study have equilibrium temperatures between 0-20°C (ASW) according to the ^{3}He/^{4}He and ^{20}Ne/^{4}He ratios. Yoshimaike, Wakutamaike and Shiraitontaki #1, however, have significantly lower ^{20}Ne/^{4}He than ASW. All these springs are located directly on the FKFZ and show similar enrichment of terrigenic He as the Ko-Fuji deep groundwater of Aoki well (^{3}He/^{4}He ~$3\cdot10^{-6}$ and ^{20}Ne/^{4}He ~2.84) which represents artesian groundwater directly from within the FKFZ (**Fig. 3f**). Remarkably, a second analysis of Shiraitonotaki (sample #2) found concentrations close to saturation, indicating that the contribution of terrigenic He is variable in time and seems – for all springs and groundwaters – to be controlled by the actual/momentary hydrogeological condition of Fuji catchment.

The most likely source of the terrigenic He can be identified by either fitting a binary mixing model to the samples or by visual analysis and comparing the identified terrigenic end-member to the typical composition of mantle and crustal He. As for the purpose of identifying potential upwelling of deep groundwater into the shallow groundwater and springs of Fuji watershed visual analysis provides sufficient detail, in **Fig. 3e** the respective mixing lines for 100%, 75%, 50%, 25% and 0% mantle He are given. Most deep thermal wells from Fuji catchment, and particularly Fujinomiya (FJM) onsen which lies almost directly below Aoki well, show terrigenic He that is isotopically lighter than the He of the subducting mantle slab of the volcanic front of Japan. This enrichment in ^{3}He therefore leads to the conclusion that MORB-type mantle He is injected into the Fuji system and its groundwaters, with the non-atmospheric He contributions being between approximately 12.5% (Wakutamaike spring) and 75% (Ko-Fuji deep groundwater and Yoshimaike spring) to the total He in the sampled waters.

Remarkably, besides MORB-type mantle He, the analysis of the light noble gases suggests an additional source of terrigenic He in the Fuji groundwater system: Despite being very close to Yoshimaike spring (<1km), Wakutamaike spring is enriched in isotopically heavy He (e.g., accumulated crustal He). Such enrichment of crustal He is also reported for the TNK onsen deep thermal well, located upstream along the FKFZ, at the base of the Misaka-Tenshu Mountain range. The available He and Ne data thus suggest that a significant portion of the water feeding Wakutamaike spring ascends through the complex network of faults and clinkers in a nearly isolated fashion from the surrounding hydrogeological system and creates a hydrogeological connection between the thermal water at the base of the Misaka-Tenshu Mountain range and Wakutamaike spring.

Although mixing of different groundwaters (i.e., Ko-Fuji deep groundwater with shallow groundwaters) appears the most likely cause for the observed distribution of noble gases around Fuji, the possibility that MORB-type mantle He is transported upwards by gases (e.g., CO_2)

which dissolve in the groundwater cannot be ruled out (see Aizawa, et al. [29]). A conclusive analysis on the transport thus calls for additional tracer investigations.

Extended results: Microbial eDNA

The eDNA-based microbial community compositions of the investigated springs and groundwaters are illustrated in **Fig. S2**. For *Bacteria* and *Archaea*, the community structure is illustrated on the phylum level for phyla that make up at least 1% of the operational taxonomic units (OTUs) (**Fig. S2, top part)**, and for *Archaea,* the community structure is furthermore illustrated on the order level for all detected OTUs (**bottom part)**. *Shannon entropy*-based alpha-diversity indices for *Bacteria* and *Archaea* (Shannon $H'_{\mathrm{B+A}}$), and *Archaea* only (Shannon H'_{A}), are also given in **Fig. S2**.

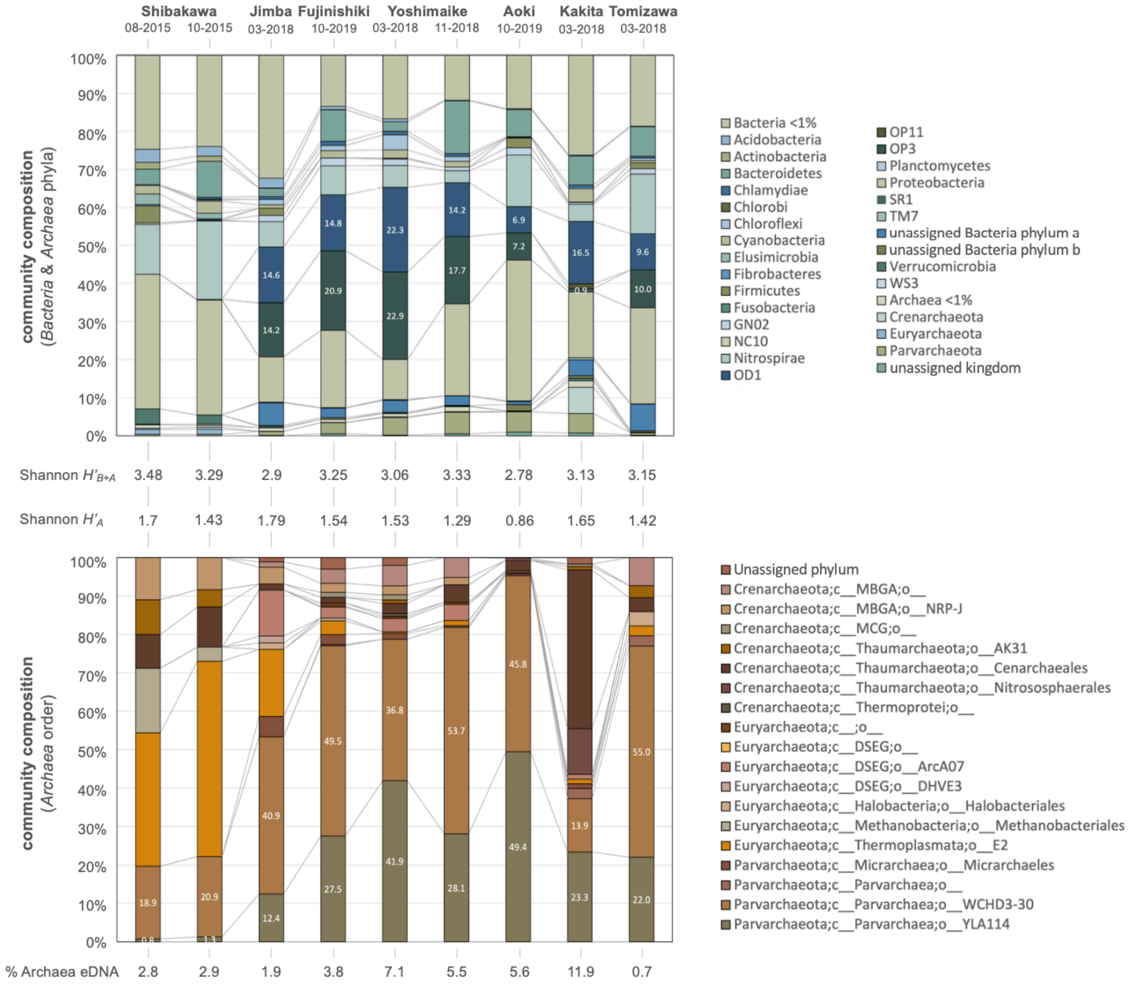

Figure S2: Relative abundance of *Bacteria* and *Archaea* operational taxonomic units (OTUs) on phylum-level considering only OTUs >1% of total eDNA (top) and *Archaea* OTUs on order-level for all identified OTUs (bottom). Indicated are also *Shannon entropy*-based alpha-diversity indices for both *Bacteria* and *Archaea* (Shannon H'_{B+A}) and only *Archaea* (Shannon H'_{A}), as well as the fraction of *Archaea* of the total prokaryotic eDNA (% *Archaea* eDNA). Shibakawa data are taken from Sugiyama, et al. [30]. All data are provided in tabular form in **Extended Data Table 2**. Abbreviations: Jimba = Jimbanotaki, Kakita = Kakitagawa.

The community structure of *Bacteria* and *Archaea* separates the microbial community in the most upstream spring (Shibakawa) from all other sites, as Shibakawa does not contain significant amounts of the OD1, OP11 and OP3 phyla. All other sites show similar community structures on the phylum level. This is reflected by H'_{B+A}, which only varies within a narrow band (between 2.78 (Aoki) and 3.48 (Shibakawa)) and does not exhibit a systematic pattern. In

contrast to *Bacteria* and *Archaea,* the community structure of *Archaea* exhibits a clear pattern: While in the upstream most spring (Shibakawa), *Archaea* of the class Parvarchaea make up only 20% of all *Archaea* OTUs, this percentage increases gradually in the downstream direction of southwestern springs and reaches a dominating 80% in Yoshimaike. Parvarchaea make up 95% of all *Archaea* in Ko-Fuji groundwater as measured in Aoki well. They are also highly important in Kakitagawa (37%), the largest and most downstream spring of the eastern subbasin, but not as dominant as in Tomizawa (77%), a spring on the foot of Mt. Ashitaka.

The Parvarchaea found in the waters of Mt. Fuji are primarily made up of the two uncultivated candidate orders YLA114 and WCHD3-30. The ultra-small, candidate extremophile order YLA114 was first detected on the hydrothermal vents of Yellowstone Lake by Kan, et al. [31] and later found in other extreme habitats such as volcanic lakes[32], hypersaline and methanogenic microbial mats[33], uranium-rich deep groundwater[34], or oxygen-depleted deep sea environments and deep hydrothermal vents[35]. They have even been suggested as key organisms in the degradation of hydrocarbons[36,37]. The candidate extremophile order WCHD3-30 was first detected by Dojka, et al. [38] in the methanogenic layers of an aquifer contaminated by hydrocarbons and chlorinated solvents, and was later found in similarly extreme environments and often alongside YLA114, such as in oxygen-depleted deep sea and hydrothermal vent environments[35] and uranium-rich deep groundwater[34]. Sugiyama, et al. [30] were the first who identified WCHD3-30 and YLA114 in Ko-Fuji groundwater (also in Aoki well), an environment which bears a strong resemblance to the confined groundwater body at 500 m depth in which Coral, et al. [34] found them to be the dominant *Archaea*. Sugiyama, et al. [30] furthermore observed that a typhoon-induced torrential rainfall event resulted in substantially increased concentrations of suspended *Archaea* in Aoki well. The concentrated recharge pulse from the torrential rainfall event thus resulted in an increase in groundwater flow velocity also through Ko-Fuji aquifer, which in turn provoked an increased detachment of microbes from the matrix[30]. While the total concentration of *Archaea* increased, the relative contributions of WCHD3-30 and YLA114 decreased, indicating that WCHD3-30 and YLA114 are primarily living in suspension rather than attached to the matrix, making both WCHD3-30 and YLA114 potential microbial tracers of upwelling of deep groundwater into shallow groundwater and springs also under average hydraulic conditions. However, the microbial data alone does not allow precluding the possibility of these Parvarchaea to have grown locally in the springs, only via additional, independent tracer data one can be certain that they are indeed signs of the admixture of upwelling Ko-Fuji deep groundwater.

With values ranging from ~750 (Tomizawa) to ~7,000 cells/mL (Wakutamaike), the total density of prokaryotes observed in the sampled springs and groundwaters of Mt. Fuji are extremely low and would normally be expected for depths of thousands rather than tens or hundreds of meters, let alone in springs[39,40]. Despite this overall interesting aspect of the total density of prokaryotic cells, a clear pattern is not identifiable in the data (see **Tab. 1 or Extended Data Table 1** for specific values).

References

1 Kamitani, T., Watanabe, M., Muranaka, Y., Shin, K.-C. & Nakano, T. Geographical characteristics and sources of dissolved ions in groundwater at the southern part of Mt. Fuji (in Japanese). *J. Geogr.* **126**, 43-71, doi:10.5026/jgeography.126.43 (2017).

2 Yasuhara, M., Kazahaya, K. & Marui, A. in *Fuji Volcano* (eds S. Aramaki, T. Fujii, S. Nakada, & N. Miyaji) 389-405 (Yamanashi Institute of Environmental Sciences, 2007).

3 Jasechko, S. Global isotope hydrogeology - Review. *Rev. Geophys.*, doi:10.1029/2018RG000627 (2019).
4 Schilling, O. S., Cook, P. G. & Brunner, P. Beyond classical observations in hydrogeology: The advantages of including exchange flux, temperature, tracer concentration, residence time and soil moisture observations in groundwater model calibration. *Rev. Geophys.* **57**, 146-182, doi:10.1029/2018RG000619 (2019).
5 Schilling, O. S. *et al.* Quantifying groundwater recharge dynamics and unsaturated zone processes in snow-dominated catchments via on-site dissolved gas analysis. *Water Resour. Res.* **57**, e2020WR028479, doi:10.1029/2020WR028479 (2021).
6 Galewsky, J. *et al.* Stable isotopes in atmospheric water vapor and applications to the hydrologic cycle. *Rev. Geophys.* **54**, 809-865, doi:10.1002/2015RG000512 (2016).
7 Kendall, C. & McDonnell, J. J. *Isotope tracers in catchment hydrology.* (Elsevier, 1998).
8 Matsuda, T. in *Fuji Volcano* (eds S. Aramaki, T. Fujii, S. Nakada, & N. Miyaji) 45-57 (Yamanashi Institute of Environmental Sciences, 2007).
9 Yaguchi, M., Muramatsu, Y., Chiba, H., Okumura, F. & Ohba, T. The origin and hydrochemistry of deep well waters from the northern foot of Mt. Fuji, central Japan. *Geochem. J.* **50**, 227-239, doi:10.2343/geochemj.2.0409 (2016).
10 Aoki, Y., Tsunematsu, K. & Yoshimoto, M. Recent progress of geophysical and geological studies of Mt. Fuji Volcano, Japan. *Earth Sci. Rev.* **194**, 264-282, doi:10.1016/j.earscirev.2019.05.003 (2019).
11 Tosaki, Y. & Asai, K. Groundwater ages in Mt. Fuji (in Japanese). *J. Geogr.* **126**, 89-104, doi:10.5026/jgeography.126.89 (2017).
12 Hayashi, T. Understanding the groundwater flow system at the northern part of Mt. Fuji: Current issues and prospects (in Japanese). *J. Geogr.* **129**, 677-695, doi:10.5026/jgeography.129.677 (2020).
13 Nagai, T., Takahashi, M., Hirahara, Y. & Shuto, K. Sr-Nd isotopic compositions of volcanic rocks from Fuji, Komitake and Ashitaka Volcanoes, Central Japan (in Japanese). *Proceedings of the Institute of Natural Sciences, Nihon University* **39**, 205-215 (2004).
14 Nakamura, H., Iwamori, H. & Kimura, J.-I. Geochemical evidence for enhanced fluid flux due to overlapping subducting plates. *Nat. Geosci.* **1**, 380-384, doi:10.1038/ngeo200 (2008).
15 Sano, Y. & Wakita, H. Distribution of 3He/4He ratios and its implications for geotectonic structure of the Japanese Islands. *J. Geophys. Res.* **90**, 8729-8741, doi:10.1029/JB090iB10p08729 (1985).
16 Koshikawa, M. K. *et al.* Using isotopes to determine the contribution of volcanic ash to Sr and Ca in stream waters and plants in a granite watershed, Mt. Tsukuba, central Japan. *Environ. Earth Sci.* **75**, 501, doi:10.1007/s12665-015-5097-9 (2016).
17 Geospatial Information Authority of Japan (GSI). Elevation tile map of Japan (DEM5A; resolution: 5m). (GSI, Tokyo, 2021).
18 Chiba, T., Kaneta, S. & Suzuki, Y. in *The International Archives of the Photogrammetry* Vol. XXXVII Ch. B2, 1071-1076 (Remote Sensing and Spatial Information Sciences, 2008).
19 Air Asia Survey Co. Ltd. *Red Relief Image Map of Japan (RRIM® 10_2016).* (Geospatial Information Authority of Japan (GSI), 2016).
20 National Institute of Advanced Industrial Science and Technology Japan (AIST). Active fault database of Japan, April 26, 2019 edition. Research information disclosure database DB095. (AIST, Tsukuba, Japan, 2019).

21 Bird, P. An updated digital model of plate boundaries. *Geochem. Geophys. Geosy.* **4**, doi:10.1029/2001GC000252 (2003).
22 Lin, A., Iida, K. & Tanaka, H. On-land active thrust faults of the Nankai–Suruga subduction zone: The Fujikawa-kako Fault Zone, central Japan. *Tectonophysics* **601**, 1-19, doi:10.1016/j.tecto.2013.04.020 (2013).
23 Van Horne, A., Sato, H. & Ishiyama, T. Evolution of the Sea of Japan back-arc and some unsolved issues. *Tectonophysics* **710-711**, 6-20, doi:10.1016/j.tecto.2016.08.020 (2017).
24 Brennwald, M. S., Schmidt, M., Oser, J. & Kipfer, R. A Portable and autonomous mass spectrometric system for on-site environmental gas analysis. *Environ. Sci. Technol.* **50**, 13455-12463, doi:10.1021/acs.est.6b03669 (2016).
25 Brennwald, M. S., Tomonaga, Y. & Kipfer, R. Deconvolution and compensation of mass spectrometric overlap interferences with the miniRUEDI portable mass spectrometer. *MethodsX* **7**, 101038, doi:10.1016/j.mex.2020.101038 (2020).
26 Kipfer, R., Aeschbach-Hertig, W., Peeters, F. & Stute, M. in *Noble gases in geochemistry and cosmochemistry* Vol. 47 *Reviews in Mineralogy and Geochemistry* (eds D. Porcelli, C. Ballentine, & R. Wieler) Ch. 14, 615-700 (2002).
27 Umeda, K. & Ninomiya, A. Helium isotopes as a tool for detecting concealed active faults. *Geochem. Geophys. Geosy.* **10**, Q08010, doi:10.1029/2009GC002501 (2009).
28 Sano, Y. & Fischer, T. P. in *The noble gases as geochemical tracers Advances in isotope geochemistry* (ed P. Burnard) Ch. 10, 249–317 (Springer, 2013).
29 Aizawa, K. *et al.* Gas pathways and remotely triggered earthquakes beneath Mount Fuji, Japan. *Geology* **44**, 127-130, doi:10.1130/G37313.1 (2016).
30 Sugiyama, A., Masuda, S., Nagaosa, K., Tsujimura, M. & Kato, K. Tracking the direct impact of rainfall on groundwater at Mt. Fuji by multiple analyses including microbial DNA. *Biogeosciences* **15**, 721-732, doi:10.5194/bg-15-721-2018 (2018).
31 Kan, K. *et al.* Archaea in Yellowstone Lake. *ISME J.* **5**, 1784-1795, doi:10.1038/ismej.2011.56 (2011).
32 Cabassi, J. *et al.* Geosphere-biosphere interactions in bio-activity volcanic lakes: Evidences from Hule and Rìo Cuarto (Costa Rica). *PLoS ONE* **9**, e102456, doi:10.1371/journal.pone.0102456 (2014).
33 Wong, H. L. *et al.* Dynamics of archaea at fine spatial scales in Shark Bay mat microbiomes. *Sci. Rep.* **7**, 46160, doi:10.1038/srep46160 (2017).
34 Coral, T. *et al.* Microbial communities associated with uranium in-situ recovery mining process are related to acid mine drainage assemblages. *Sci. Total Environ.* **628-629**, 26-35, doi:10.1016/j.scitotenv.2018.01.321 (2018).
35 Xia, X., Guo, W. & Liu, H. Basin scale variation on the composition and diversity of Archaea in the pacific ocean. *Front. Microbiol.* **8**, 2057, doi:10.3389/fmicb.2017.02057 (2017).
36 Shelton, J. L. *et al.* Environmental drivers of differences in microbial community structure in crude oil reservoirs across a methanogenic gradient. *Front. Microbiol.* **7**, 1535, doi:10.3389/fmicb.2016.01535 (2016).
37 Bruchberger, M. C. *et al.* Biodegradability of legacy crude oil contamination in Gulf War damaged groundwater wells in Northern Kuwait. *Biodegradation* **30**, 71-85, doi:10.1007/s10532-019-09867-w (2019).
38 Dojka, M. A., Hugenholtz, P., Haack, S. K. & Pace, N. R. Microbial diversity in a hydrocarbon- and chlorinated-solvent-contaminated aquifer undergoing Intrinsic bioremediation. *Appl. Environ. Microb.* **64**, doi:10.1128/AEM.64.10.3869-3877.1998 (1998).

39 Magnabosco, C. *et al.* The biomass and biodiversity of the continental subsurface. *Nat. Geosci.* **11**, 707-717, doi:10.1038/s41561-018-0221-6 (2018).
40 Sugiyama, A., Tsujimura, M. & Kato, K. Issues and Perspectives on Environmental Microbial Dynamics and Groundwater Flow System Research (in Japanese). *J. Groundw. Hydrol.* **62**, 431-448, doi:10.5917/jagh.62.431 (2020).
