# Supplementary material for: Revisiting Mt Fuji’s groundwater origins with helium, vanadium and environmental DNA tracers
Source: Nat Water. 2023 Jan 19;1(1):60–73. doi: 10.1038/s44221-022-00001-4 (PMC11041693; doi:10.1038/s44221-022-00001-4)
Supplement: Supplementary file 2 — Reporting Summary [file 44221_2022_1_MOESM2_ESM.pdf]

nature portfolio

Corresponding author(s): Oliver S. Schilling

Last updated by author(s): Sep 28, 2022

Reporting Summary

Nature Portfolio wishes to improve the reproducibility of the work that we publish. This form provides structure for consistency and transparency in reporting. For further information on Nature Portfolio policies, see our Editorial Policies and the Editorial Policy Checklist.

Statistics

For all statistical analyses, confirm that the following items are present in the figure legend, table legend, main text, or Methods section.

n/a	Confirmed	
☐	☒	The exact sample size (*n*) for each experimental group/condition, given as a discrete number and unit of measurement
☐	☒	A statement on whether measurements were taken from distinct samples or whether the same sample was measured repeatedly
☒	☐	The statistical test(s) used AND whether they are one- or two-sided<br>*Only common tests should be described solely by name; describe more complex techniques in the Methods section.*
☐	☒	A description of all covariates tested
☐	☒	A description of any assumptions or corrections, such as tests of normality and adjustment for multiple comparisons
☐	☒	A full description of the statistical parameters including central tendency (e.g. means) or other basic estimates (e.g. regression coefficient) AND variation (e.g. standard deviation) or associated estimates of uncertainty (e.g. confidence intervals)
☒	☐	For null hypothesis testing, the test statistic (e.g. *F*, *t*, *r*) with confidence intervals, effect sizes, degrees of freedom and *P* value noted<br>*Give P values as exact values whenever suitable.*
☒	☐	For Bayesian analysis, information on the choice of priors and Markov chain Monte Carlo settings
☒	☐	For hierarchical and complex designs, identification of the appropriate level for tests and full reporting of outcomes
☒	☐	Estimates of effect sizes (e.g. Cohen's *d*, Pearson's *r*), indicating how they were calculated

Our web collection on statistics for biologists contains articles on many of the points above.

Software and code

Policy information about availability of computer code

Data collection	Mozilla Firefox v.68-v98; QGIS v3.6-v.3.16
Data analysis	Microsoft Office (Excel + Powerpoint) 2016 for Mac; QGIS v3.6 -v3.16; QIIME 2; R v3.6.2 + phyloseq package v1.30.00; Python v3.8 + ruediPy package v2019

For manuscripts utilizing custom algorithms or software that are central to the research but not yet described in published literature, software must be made available to editors and reviewers. We strongly encourage code deposition in a community repository (e.g. GitHub). See the Nature Portfolio guidelines for submitting code & software for further information.

Data

Policy information about availability of data

All manuscripts must include a data availability statement. This statement should provide the following information, where applicable:

- Accession codes, unique identifiers, or web links for publicly available datasets
- A description of any restrictions on data availability
- For clinical datasets or third party data, please ensure that the statement adheres to our policy

All data used in this study are compiled into Extended Data Tables 1 & 2, which are supplied as supporting information alongside this manuscript and stored on the public data repository HydroShare under doi: 10.4211/hs.4eac370d12e142b5aa718e5deb57da39 (Schilling, et al. 159). The nucleotide sequence datasets obtained in this study were filed in the DNA Databank of Japan (DDBJ) under accession number DRA013474.

Human research participants

Policy information about studies involving human research participants and Sex and Gender in Research.

Reporting on sex and gender	N/A
Population characteristics	N/A
Recruitment	N/A
Ethics oversight	N/A

Note that full information on the approval of the study protocol must also be provided in the manuscript.

Field-specific reporting

Please select the one below that is the best fit for your research. If you are not sure, read the appropriate sections before making your selection.

☐ Life sciences ☐ Behavioural & social sciences ☒ Ecological, evolutionary & environmental sciences

For a reference copy of the document with all sections, see nature.com/documents/nr-reporting-summary-flat.pdf

Ecological, evolutionary & environmental sciences study design

All studies must disclose on these points even when the disclosure is negative.

Study description	Hydrogeological, hydrochemical and microbiological study of the water origins of different springs and groundwater wells within Mt. Fuji watershed based on a combined field and meta study. The study reveals previously unknown and significant deep groundwater circulation and upwelling of deep groundwater into groundwater wells and springs along the Fujikawa Kako fault zone. While classic hydrogeological tracer methods previously employed at Fuji watershed did not reveal this, the combined study of helium, vanadium and eDNA in the different waters around Fuji allowed an unambiguous identification of these deep groundwater water fluxes. The final dataset constructed for the analysis contains approximately 9,500 data points from over 350 different sites.
Research sample	The analysed data consist of a) field-sampled groundwater physico-chemical and hydrochemical parameters (water temperature, electrical conductivity, pH, major ions concentrations, stable water isotope ratios, dissolved gas concentrations and isotope ratios, trace element concentrations), and b) microbial DNA present in the water samples (i.e., microbial eDNA). As this is a combined field and meta study, detailed information on sampling and sample analysis is provided only for the sampled and analysed field measurements, and information for the data obtained from literature, are provided in the main manuscript.
Sampling strategy	This is a combined field and meta study. All data that we obtained from our own measurements and that we could find in scientific literature and in other publicly accessible sources were combined into one large dataset. The sampling strategy thus was to obtain as much groundwater and spring water data as possible from as many distributed sites as possible within Mt. Fuji catchment, subject to the constraints given by time, territorial accessibility, funding and personnel.
Data collection	The database consists of a mix of data obtained from samples taken in the field and analyzed on site and in the laboratory by the authors themselves as well as of data collected from the national hydrological database of Japan, published scientific literature and publicly available data sources (e.g., governmental reports, mineral water company water quality reports, hot spring resorts water quality reports)
Timing and spatial scale	The data covers samples taken from springs and groundwater wells scattered throughout the entire 100 km2 Mt.Fuji catchment, spanning a period of 41 years (1979-2020).
Data exclusions	No data were excluded
Reproducibility	No laboratory or natural experiments were conducted. All data were sampled directly from the natural environment. The entire database collected and used for this study is given in the Extended data tables 1 + 2, which are supplied alongside the manuscript as well as on the HydroShare repository stated in the data availability statement. All software used is reported in the manuscript, the code availability statement as well as the reporting summary.
Randomization	Not relevant for this environmental study covering data obtained from locations scattered throughout the entire Mt. Fuji catchment.
Blinding	Not relevant for this environmental study covering data obtained from locations scattered throughout the entire Mt. Fuji catchment.

Did the study involve field work? ☒ Yes ☐ No

Field work, collection and transport

Field conditions	Field work was conducted under average conditions and such that environmental conditions (e.g., extreme temperatures, extreme weather, extreme discharge) were not impacting the results. Water samples were taken from undisturbed and freely flowing water sources. All relevant physico-chemical information of the obtained water samples are provided directly in the supplied dataset or described in the original literature from which the already published data were obtained from. All data and references are provided in the complete dataset provided alongside the paper.
Location	All sampling locations and dates are given precisely in the complete dataset provided alongside the paper.
Access & import/export	Data were collected and analyzed directly on site in Japan or in laboratories in Japan (most data) or in Switzerland (only 4 water samples for high resolution dissolved noble gas isotope analysis). Access and import/export followed general local regulations and did not require any permits. For data obtained from private groundwater wells and springs, permissions from the owners were obtained prior to sampling. Detailed information on sampling, laboratory analysis and location of analysis are provided in the paper's methods section for samples obtained and analyzed by the authors in this study. Information on data obtained from literature are provided in the original literature.
Disturbance	No disturbances were caused.

Reporting for specific materials, systems and methods

We require information from authors about some types of materials, experimental systems and methods used in many studies. Here, indicate whether each material, system or method listed is relevant to your study. If you are not sure if a list item applies to your research, read the appropriate section before selecting a response.

Materials & experimental systems

n/a	Involved in the study
☒	☐ Antibodies
☒	☐ Eukaryotic cell lines
☒	☐ Palaeontology and archaeology
☒	☐ Animals and other organisms
☒	☐ Clinical data
☒	☐ Dual use research of concern

Methods

n/a	Involved in the study
☒	☐ ChIP-seq
☒	☐ Flow cytometry
☒	☐ MRI-based neuroimaging
